# Targeting MAO-B with Small-Molecule Inhibitors: A Decade of Advances in Anticancer Research (2012–2024)

**DOI:** 10.3390/molecules30010126

**Published:** 2024-12-31

**Authors:** Iyman Alsaad, Diana M. A. Abdel Rahman, Ola Al-Tamimi, Shayma’a A. Alhaj, Dima A. Sabbah, Rima Hajjo, Sanaa K. Bardaweel

**Affiliations:** 1 Department of Pharmaceutical Sciences, School of Pharmacy, University of Jordan, Amman 11942, Jordanolatameemi@gmail.com (O.A.-T.);; 2 Department of Pharmacy, Faculty of Pharmacy, Al-Zaytoonah University of Jordan, P.O. Box 130, Amman 11733, Jordan; dima.sabbah@zuj.edu.jo (D.A.S.); r.hajjo@zuj.edu.jo (R.H.); 3 Laboratory for Molecular Modeling, Division of Chemical Biology and Medicinal Chemistry, Eshelman School of Pharmacy, The University of North Carolina at Chapel Hill, Chapel Hill, NC 27599, USA; 4 Jordan CDC, Amman 11118, Jordan

**Keywords:** cancer, monoamine oxidase B (MAO-B), MAO-B inhibitors, MAO-B network biology, MAO-B ligand interactions, anticancer agents

## Abstract

Monoamine oxidase B (MAO-B) is a key enzyme in the mitochondrial outer membrane, pivotal for the oxidative deamination of biogenic amines. Its overexpression has been implicated in the pathogenesis of several cancers, including glioblastoma and colorectal, lung, renal, and bladder cancers, primarily through the increased production of reactive oxygen species (ROS). Inhibition of MAO-B impedes cell proliferation, making it a potential therapeutic target. Various monoamine oxidase B inhibitors have shown promise in inhibiting tumor growth and inducing apoptosis across different cancer types. In this review, we investigate MAO-B network biology, which highlighted glycolysis pathways as notable links between MAO-B and cancer. Further molecular modeling analysis illustrated the basis of MAO-B ligand binding, revealing a hydrophobic binding pocket, with key residues such as Tyr398 and Tyr435 playing crucial roles in substrate oxidation. MAO-B inhibitors that were reportsed in the literature (2012–2024) and their potential application in cancer therapy were discussed, highlighting key molecular scaffolds, such as propargyl analogs of phenyl alkyl amines, hydrazine derivatives, cyclopropylamine derivatives, MAO-B activated pro-drugs, and natural phenylpropanoid derivatives. The reported literature underscores the therapeutic potential of MAO-B inhibitors as versatile anticancer agents, warranting further investigation to optimize their efficacy and specificity across various malignancies.

## 1. Introduction

Monoamine oxidase (MAO), also referred to as tyramine oxidase, is a FAD-dependent enzyme that is located at the mitochondrial outer membrane [1,2]. It catalyzes the oxidative deamination of biogenic amines such as monoamine neurotransmitters (serotonin, dopamine, and norepinephrine) and dietary amines [2,3,4,5]. MAO enzymes have the covalently bound flavin adenine (FAD) cofactor that is essential for their enzymatic activity [3]. There are two isoforms of monoamine oxidase enzymes: monoamine oxidase A (MAO-A) and monoamine oxidase B (MAO-B). These isoforms differ in tissue distribution and cell type localization, enzyme activity, substrate specificity, and their catalysis kinetics [2,4,6]. Typically, MAOs are found in neurons and astroglia cells within the central nervous system (CNS) and in other organs. MAO-A is expressed in the gastrointestinal tract, lungs, liver, and placenta, while MAO-B is found in platelets [5]. *MAO-A* and *MAO-B* genes have 15 exons possessing identical intron-exon organization attained by duplication of a common ancestral gene; hence, they are termed isoenzymes [4,7]. Both genes are located on X-chromosomes organized in opposite directions, tail to tail 24 kb apart [4]. Moreover, their promoter regions share 60% sequence homology with distinct organization of transcription elements. The *MAO-B* gene has a molecular mass of 58.8 kD and it encodes a 520-amino acid protein [1,2].

*MAO-B* promoter is regulated by transcription factors Sp1/Sp4/Sp3 binding sites. Transcription factors Sp1 and Sp4 can activate the *MAO-B* promoter, while activation can be suppressed by the overexpression of Sp3 [6,8]. Sp1 levels are found to be elevated in gliomas, which was reported to have an adverse relationship with patient survival in gliomas [8]. Furthermore, the pentapeptide sequences: cyst-tyr-gly-gly-ser, are shared in both *MAO-A* and *MAO-B* enzymes. The cysteine397 is incorporated in this pentapeptide and is covalently bound by the FAD cofactor via a thioester bond [2,9,10]. MAO-B enzyme is attached to the outer mitochondrial membrane via a transmembrane α-helix allocated inside the carboxyl-terminal domain, with supplementary membrane interactions occurring with another hydrophobic residue. MAO-B forms a homodimer in which each subunit is 59,000 Dalton [1,2].

Targeting MAO-B with small-molecule inhibitors has emerged as a promising frontier in anticancer drug discovery. Experimental research revealed the role of MAO-B in generating ROS by oxidative deamination activity may create a suitable microenvironment for tumor initiation and progression [11]. Overexpression of MAO-B in many types of cancer, such as colorectal, glioblastoma, breast, lung, pancreatic, and liver cancer, emphasized the proposed linkage. A wide range of MAO-B inhibitor scaffolds have been reported as potential anticancer agents with an evident ability to modulate cellular growth and apoptosis in the preclinical setting [11,12,13,14,15]. Various derivatives, including phenyl alkyl amine, cyclopropylamine, hydrazine, phenylpropanoid, pyridine derivatives linked to nitrogen mustard, polyamine-based compounds, hydrathiazole, Chalcone, and chromone derivatives, each contribute differently to anticancer activity [5,11,16,17,18,19,20]. For example, selegiline and rasagiline have demonstrated effectiveness in reducing prostate cancer cell viability and enhancing the efficacy of chemotherapeutic agents. At the same time, pargyline and tranylcypromine have shown efficacy against breast and prostate cancers by inducing apoptosis and cell cycle arrest. A few inhibitors reached phase I and phase II clinical trials and succeeded in improving cancer treatment outcomes [17,21,22,23,24,25].

In this context, we highlight the relevance of MAO-B’s systems biology and metabolic interactions in cancer development and progression. A systematic search was conducted to identify human MAO-B’s nearest neighbor (NN) proteins, which were analyzed for functional interactions and pathway enrichment. Molecular modeling studies further confirmed the structural basis of MAO-B ligand interactions and provided important insight for drug discovery efforts. Finally, we discuss MAO-B inhibitors that have demonstrated anticancer activity in both preclinical and clinical studies.

### MAO-B Catalysis

**Figure 1:**
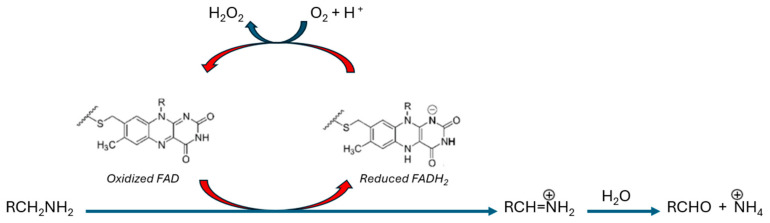
Oxidation of amines into their corresponding imine by MAO-B-bound FAD, followed by non-enzymatic hydrolysis to their respective aldehydes.

MAOs carry out the oxidation of amines (primary, secondary, and tertiary), involving multiple neurotransmitters, into the respective imines. MAO-A catalyzes serotonin, norepinephrine, and dopamine metabolism, whereas MAO-B favorably catalyzes the metabolism of benzylamine, phenethylamine, and other catecholamines [3]. The oxidized products are hydrolyzed non-enzymatically into the corresponding aldehydes or ketones, with concurrent production of reactive oxygen species (ROS) [2]. The resulting aldehydes are rapidly metabolized into their corresponding acid by aldehyde dehydrogenase (ALDH), with hydrogen peroxide (H_2_O_2_) and ammonia (NH_3_) as by-products of the MAOs’ catalytic reactions [2,3]. This catalytic cycle occurs in two steps (Figure 1). Firstly, the reduction of FAD (flavin adenine dinucleotide) into FADH_2_ (dihydro-flavin adenine dinucleotide) is accompanied by the oxidation of the amine neurotransmitter (R–CH_2_-NH_2_) with the production of the respective imine. Secondly, oxygen (O_2_) oxidizes the cofactor FAD by accompanying the production of hydrogen peroxide (H_2_O_2_) [2].

## 2. MAO-B and Cancer

MAO-B overactivity results in an escalated production of damaging species such as aldehydes and hydrogen peroxide, which promotes oxidative and inflammatory stress development. This scenario is correlated to pathogenesis and numerous disease progressions such as neurodegenerative diseases, cardiovascular disorders, and cancer [3,5].

Recently, MAO-B inhibitors have grabbed the attention of several types of cancer treatments due to an evidenced correlation between MAO-B overexpression and several cancers, such as glioblastoma and colorectal cancer [2]. Additionally, MAO-B levels are correlated with higher cancer recurrence rates and poor prognosis. For instance, a significant decline in prostate-specific antigen in patients with recurring castrate-sensitive prostate cancer following treatment by irreversible MAOB inhibitor phenelzine [2,3,5].

MAO-B catalysis is a major source of ROS, which has a dual action in cancer development. ROS promotes oxidative-stress-induced cell death. On the other hand, ROS promotes resistance to hypoxia, cellular transformation, proliferation, angiogenesis, and metastasis [26]. Cancer cells often produce higher levels of reactive oxygen species (ROS), which can promote proliferation and survival by activating signaling pathways. However, elevated ROS levels also pose a risk of oxidative damage that could lead to cell death [3,27]. To manage this, cancer cells upregulate their antioxidant systems to maintain ROS homeostasis and prevent lethal oxidative stress. The shift in the redox environment in cancer cells makes them more dependent on their antioxidant systems [3,27,28]. This has led to therapeutic strategies that aim to disrupt this balance either by inhibiting ROS production or by disabling antioxidant defenses, which may lead to increased oxidative stress and cell death in cancer cells [3,27].

### 2.1. Bladder Cancer

Resta et al. reported that MAO-B is present in human urothelial tumor explants and the bladder cancer (BC) cell line AY27. Selective inhibition of MAO-B restricted the formation of mitochondrial ROS, the progression of the BC cells’ cell cycle, and their proliferation [29]. For instance, the treatment of AY27 cells by using selective MAO-B inhibitors (deprenyl) had interestingly a redundant effect on AY27 cells after only 4 days in culture [29]. In a study examining the role of monoamine oxidase B (MAO-B) in BC, researchers analyzed sixty formalin-fixed paraffin-embedded bladder samples from patients (mean age 65) [30]. Immunohistochemical analysis revealed positive MAO-B immunoreactivity across all histological stages. Notably, MAO-B levels were significantly higher in high-grade pTa and pT2 groups compared to low-grade pTa (*p* < 0.01). These findings indicate that MAO-B may play a crucial role in BC pathogenesis and has potential as a biomarker for clinical response and prognosis [30].

### 2.2. Glioma

MAO-B has been found overexpressed in several malignancies in recent years, chiefly in gliomas and human glioblastoma [31]. Sharpe et al. (2016) revealed that the levels of MAOB were, on average, 8 times higher in gliomas than in control tissue [8]. Gliomas (malignant brain tumors) are ranked from grade one to grade four by the World Health Organization (WHO), where grade four glioma has the worst prognosis. Gliomas are distinguished by their quick growth and expansion into surrounding tissues. In human gliomas, MAO-B activity is also observed to be markedly elevated [8,11,31]. Accordingly, a strong association between MAO-B overexpression and high-grade glioma has been reported; hence, MAO-B is considered a potential target for the development of innovative anticancer therapies for glioma [11,31].

In gliomas associated with the expression of hypoxia-inducible factor 1 (HIF-1α), an overexpression of MAO-B has been observed [32]. HIF-1α induces the transcription of numerous hypoxia-responsive genes, including those encoding vascular endothelial growth factor (VEGF) and its receptors [8]. Elevated levels of nuclear-localized HIF-1α are found in most glioblastomas and anaplastic astrocytomas, particularly near necrotic areas in glioblastomas. The expression levels of these proteins are correlated with the grade of glioma tumors [8].

Additionally, nuclear factor kappa B subunit 1 (NF-kB) is believed to be the primary cause of gliomas’ resistance to radiation therapy [2]. In this regard, it has been documented that H_2_O_2_ generated by MAO-B metabolism sparks a cascade of signals that activate NF-kB. MAO-B overexpression can be linked to radiation therapy success. The finding that MAO-B is present in lung cancer cells appears to corroborate this finding, designating this enzyme as a tumor biomarker [2].

### 2.3. Colorectal Cancer

A study by Battaglin et al. (2022) mentioned that the spreading and development of tumors are caused by differentiated MAO expression, which has been detected in several cancer types [33]. It was also stated that higher levels of MAO-B were associated with worse clinical stages, more recurrences, and lower survival rates in colorectal cancer (CRC) [33]. It was also concluded that the brain-gut axis (BGA), which is a complex bidirectional signal transmission system that connects the enteric nervous system (ENS), the CNS, and the endocrine-immune system, has a crucial role in the development and carcinogenesis of colorectal cancer [33]. Interestingly, an increasing amount of data on the important roles played by several neurotransmitters and neural factors in the development of CRC may provide new perspectives that call for specialized research to clarify the fundamental mechanism underlying this tumorigenic activity [33,34]. A study by Yang et al. 2020 with 203 colorectal cancer cases revealed that, in comparison to normal tissue, MAO-B was overexpressed significantly in tumor CRC tissues (Z score = 4.01) in CRC [35]. Moreover, Yang et al. 2020 pointed out that in 177 CRC cases with advanced stages (stages II, III, and IV), MAO-B showed higher expression levels than those in stage 1 [35]. Additionally, high MAOB expression was strongly correlated with poor disease-specific survival (*p* = 0.001) and disease-free survival (*p* = 0.014) [35].

### 2.4. Liver Cancer

A study by Fan et al. (2021) has shown that liver fibrosis may result in liver cancer and cirrhosis, which could be fatal or seriously impair liver function [36]. MAO-B can act as a biomarker in the initial stages of fibrosis, which aids in the early diagnosis of liver fibrosis [36]. Using a readily manufactured probe called BiPhAA, a two-photon fluorescence imaging technique for the in vivo detection of MAO-B activity counts was developed. Additionally, the dynamic detection of endogenous MAO-B level variations in hepatic stellate cells (LX-2) was made possible by BiPhAA [36]. A study by Tabata et al. 2020 has demonstrated that in human hepatoma cells, MAO-B plays a key role in converting geranylgeraniol (GGOH) to geranylgeranoic acid (GGA), and inhibiting or downregulating MAO-B activity using inhibitors or small interfering RNAs (siRNAs) reduced the intracellular level of GGA in these cells [37]. Accordingly, maintaining normal MAO-B expression is crucial for sustaining GGA levels in human hepatocytes, which is significant as GGA displays anti-tumor properties [37].

### 2.5. Lung Cancer

MAO-B was reported to be overexpressed in non-small cell lung cancer (NSCLC) [4]. The compound known as Danshensu, which is derived from traditional oriental medicine, binds directly to MAO-B at the typical docking site as MAO-B inhibitors such as selegiline [12]. A study by Shih, 2018b has shown that at doses up to 50 μM, Danshensu suppresses MAO-B activity without compromising cell viability. The study has also shown that Danshensu has been demonstrated to improve NSCLC cells’ radiosensitivity; consequently, patients with lung cancer may benefit from MAO-B inhibitors or MAO-B-activated prodrugs [4]. A study by Kery and Papandreou (2020) has demonstrated that a major challenge in treating lung cancer is the high resistance to ionizing radiation, which diminishes the effectiveness of radiation therapy [38]. In addition, lung cancer cells overexpress MAO-B in contrast to normal cells [39]. A study by Zhang et al. (2020) has shown that *MAO-B* is one of the clinically important metabolism-related genes that can be considered a biomarker for lung adenocarcinoma diagnosis, monitoring, and prognosis [40]. For instance, MAO-B overexpression at mRNA and protein levels in NSCLC cells (A549 and NCI-H1299 cells) in response to ionizing radiation (IR) treatment was noticed in a dose-dependent manner. Hence, MAO-B can be considered a biomarker for NSCLC and IR resistance [12].

### 2.6. Kidney Cancer

One of the world’s top 10 cancers that affect adults is renal cell carcinoma (RCC). More than 75% of cases of RCC are clear cell renal cell carcinoma (ccRCC), the most common histological kidney cancer subtype [41]. MAO-B has been reported to be expressed in renal cancer [39]. A study by Hodorová et al. (2018) was centered on the immunohistochemistry evaluation of MAO-B in RCC. On the other hand, other reports demonstrated that MAO-B expression was not significantly involved in the development of renal carcinoma; MAO-B was only present in 19% of kidney tumors, and there was little positive protein expression. Furthermore, it has been shown that the loss of this enzyme in RCC results from cancer cells replacing healthy tissue. Conversely, it is conceivable that severe pathological processes in the kidney could be linked to the absence of MAO-B expression [42].

## 3. MAO-B Protein-Protein Functional Interactions Network

**Figure 2:**
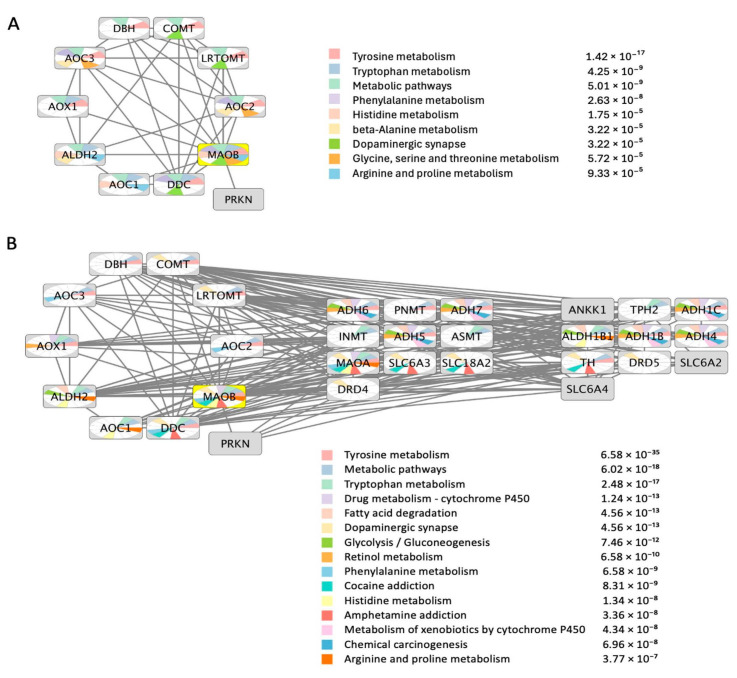
MAO-B protein-protein interaction networks. (**A**) Direct interactions network created using nearest neighbor proteins/genes. (**B**) An expanded interactions network was created by allowing the addition of 20 more seed nodes to the direct interactions network. The nodes are color-coded using a split pie chart coloring scheme indicating pathway contribution to each node from the topmost significant KEGG enrichments.

A systematic search for human MAO-B’s nearest neighbor (NN) proteins was conducted in Cytoscape version 3.10.1 [43]. The STRING App version 2.2.0 was used to identify MAO-B functional NN proteins shown in Figure 2, at a confidence level of 0.5. Details about STRING database content and confidence levels are found on the STRING website and related database publications [44]. MAO-B and its functional NN proteins were also used as a query for pathway enrichment analysis in Cytoscape. The top enrichment results are shown in Figure 2, while the complete lists of enriched pathways are provided in Tables S1 and S2.

It is notable that the top enriched pathways in the direct interactions network (Figure 2A) are mainly metabolic pathways involved in normal physiological processes, and none is clearly linked to cancer. However, these pathways are relevant to both normal and cancer cells, although cancer cells are characterized by increased amino acid uptake, altered catabolism, and enhanced biosynthesis, all of which are geared toward supporting rapid growth, survival under stress, and maintaining cellular functions in the face of metabolic challenges.

Repeating the enrichment analysis after expanding the direct interactions network by adding 20 additional interacting proteins resulted in a new set of enriched pathways, including glycolysis/gluconeogenesis, fatty acid degradation, chemical carcinogenesis, retinol metabolism, and others. All these pathways are relevant to cancer, particularly since there is an important link between glycolysis and tumorigenesis since many cancers (e.g., colorectal, glioblastoma, breast, lung, pancreatic, head, and neck) undergo a metabolic shift toward glucose-dependent metabolism to produce lactate in the presence of oxygen, which contributes to the malignant phenotype [29]. According to “Warburg theory” [45], this metabolic shift allows cancer cells to thrive in low-oxygen environments and supports rapid cell proliferation. Glycolysis was the top third enriched pathway by MAO-B’s expanded protein-protein functional interactions network, which could potentially link MAO-B to cancer pathways through glycolysis. It is known that MAOs are implicated in ROS-induced tumorigenesis [46]. ROS is known to upregulate glycolysis in cancer cells, leading to the Warburg effect. Furthermore, a recent study showed that MAO inhibitors decreased the expression of proteins involved in glucose transport (GLUT1) and transformation (HK2) [29].

## 4. The Structural Basis of MAO-B Ligand Interactions in the Binding Pocket

**Figure 3:**
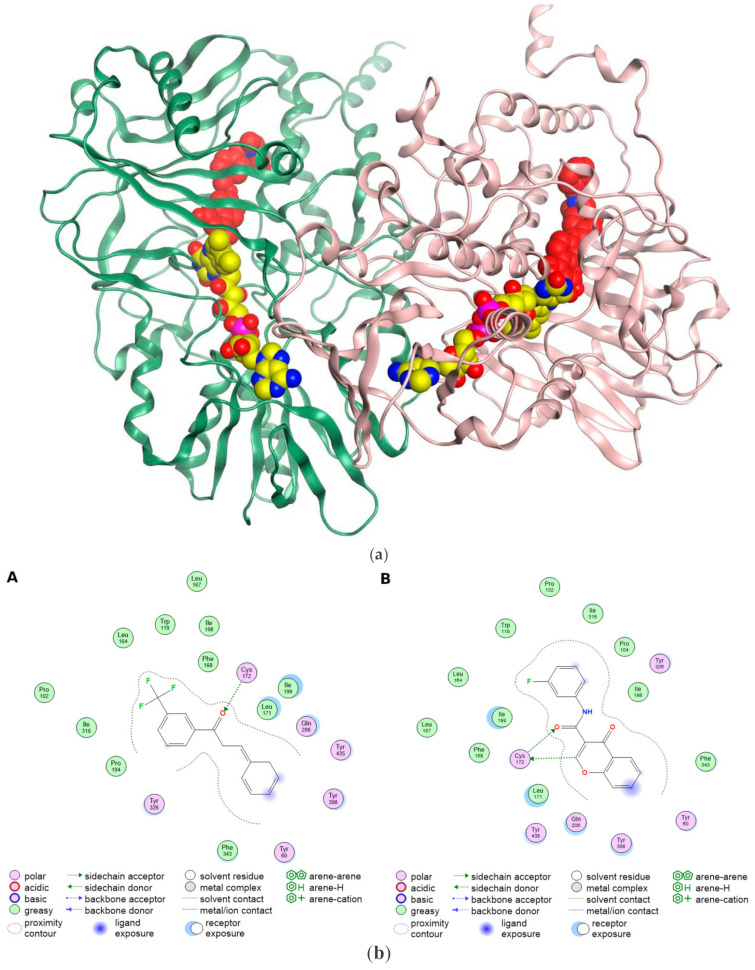
(**a**) The crystal structure of MAO-B dimer (PDB ID: 7P4F) [59]. Each monomer harbors FAD (yellow color) and co-crystallized inhibitor (5IK) (red color). Picture captured by MOE software version 2022.02 [60]. (**b**) Binding sites of (**A**) 7B0V accommodating the co-crystallized ligand (SKB) and (**B**) 6FWC accommodating (E98). Picture visualized by MOE [60].

The crystal structure of MAO B with 520 residues discloses a dimeric enzyme [47]. Each monomer embeds a water-exposed spherical structure (residues 1–488) attached to the membrane through a hydrophobic motif of the C-terminal α-helix (residues 489–500) [47]. The water-exposed part of the protein is similar to the ensembles of other flavin-dependent enzymes harboring a flavin (FAD)-binding domain and a substrate-binding domain (Figure 3a) [48]. Each MAO-B monomer contains an elongated loop (residues 461–488) that emerges from the FAD-binding domain and anchors the MOA-B backbone to the C-terminal α-helix [47]. The conformation of this loop is maintained through binding interactions between the substrate and FAD with the backbone of MAO-B [47]. This loop launches the C-terminal hydrophobic helix that diverges perpendicularly from each monomer [47]. The side chain of Arg494 furnishes ionic interaction with the charged head groups of the membranous phospholipids [47]. The substrate-binding domain encloses some hydrophobic sites (Pro109–Ile110, Trp157) that are close to the C-terminal helix and consequently contribute to membrane binding [47]. Residues 99–112 form a loop that serves as a gate to the active site; this loop might be engaged in the membrane [47]. Studies reported that MAO-B encloses three functional domains the entrance cavity, substrate pocket, and “aromatic cage” [49,50]. The entrance cavity and substrate pocket are hydrophobic [51]. Both pockets are separated by a “loop” of four amino residues: Phe168, Leu171, Ile199, and Tyr326 [47].

The binding site of MAO-B is hydrophobic, enclosed by hydrophobic and aromatic residues [19]. Water-mediated H-bond is noticed between Lys296 and the N5 atom of the cofactor flavin [52]. Tyr398 and Tyr435 are situated on opposing faces of covalently bound ligands and substrates, forming an “aromatic cage” [53,54,55]. Furthermore, the aberration of these residues changes the activity [53,54,55]. Tyr398 and Tyr435 induce the nucleophilicity of the amine substrate and assist the orientation of the substrate to facilitate its oxidation [56]. The MAO-B binding pocket is small and designated as the “entrance cavity”; rotation of Ile199 enhances the fusion of the two hydrophobic cavities (entrance (290 Å^3^) and substrate pocket (490 Å^3^)) into one big active site cavity of 700 Å^3^ [48]. Modeling studies revealed that selective MAO-B inhibitors bind with Tyr326 which is situated close to the entrance and substrate pocket [57]. The aromatic ring of Tyr326 serves as a wall in the substrate domain, leading to steric constraint in the MAO-B active site [58].

In order to explore the binding interactions of MAOB inhibitors in the MAOB binding site, we retrieved MAOB coordinates (PDB IDs: 7B0V [19] and 6FWC [20]) from the PDB repository. The binding site of 7B0V and 6FWC encloses Tyr60, Pro102, Pro104, Trp119, Leu164, Leu167, Phe168, Leu171, Cys172, Ile198, Ile199, Gln206, Ile316, Phe343, Tyr398, and Y435 (Figure 3b). Hydrophobic and polar residues encircle the binding sites and engage with ligands. Generally, the acidic and basic residues provide ionic (electrostatic) bonding while the hydrophilic residues furnish hydrogen bonding, ion-dipole, and dipole-dipole interactions. The aromatic and hydrophobic residues afford π-π stacking and van der Waals interactions, respectively. Figure 3b displays that the co-crystallized ligands SKB and E98 form an H-bond with Cys172. Furthermore, the hydrophobic and aromatic residues surround the hydrophobic and aromatic motifs, whereas the polar residues border the polar functionalities. Eventually, the fingerprints of SKB and E98 match the surrounding residues, anticipating potential affinity to induce the biological activity.

## 5. MAO-B Inhibitors as Anticancer Agents

MAO-B inhibitors have emerged as potential anticancer agents since they can modulate cellular growth and apoptosis [39]. A key feature of MAO-B inhibitors is the wide range of scaffolds contributing to their activity and specificity [61]. This includes the propargyl analog of phenyl alkyl amine, hydrazine derivative, cyclopropylamine derivative, hydrazothiazole derivative, phenylpropanoid derivative, and polyamine derivatives [5]. Each of these scaffolds contributes in different ways to the development of effective and targeted anti-cancer therapies (Table 1).

### 5.1. Phenyl Alkyl Amine Derivatives

Selegiline (**1**), the R-enantiomer of deprenyl (phenyl-isopropyl-methyl propargylamine), was synthesized in 1962 to produce a psychic revitalizer based on the recognized antidepressant effect of the known MAO inhibitors at that time. Selegiline’s early pharmacological and clinical studies showed many distinctive attributes unlike those of the formerly used MAO inhibitors, and this led to the discovery of MAO-B, the other distinct isoform for MAO [62].

Selegiline inhibits irreversibly and selectively MAO-B, causing an increment in the level of biogenic amines, particularly dopamine, and so exhibiting an antidepressant effect [63]. It also reduces the level of various neurotoxins and oxidative stress promoters; this neuroprotective effect is behind its clinical use for the treatment of Parkinson’s disease [64].

In addition, long-term treatment with selegiline increases the activity of superoxide dismutase (SOD), an important antioxidant defense in nearly all living cells exposed to oxidative injury [65]. It was also demonstrated that selegiline can prohibit apoptosis, triggered by serum shortages, glutathione deprivation, and toxins [66]. The former findings guided scientists to evaluate a suspected anticancer effect of selegiline, which was established later by its ability to prevent p53-dependent apoptosis and loss of mitochondrial function via regulating a specific set of genes [12].

Rasagiline (**2**) *(N*-propargyl-1(R)-aminoindane) is a more potent analog to selegiline; the racemic form of rasagiline was invented in the early 1970s and patented as an antihypertensive agent [67]. The R-enantiomers were isolated after that and revealed its potent irreversible and selective MAO-B inhibitory activity correlated to its dopamine-enhancing effect and neuroprotective activity that is compatible with the treatment of Parkinson’s disease [16].

**Table 1:** Overview of molecular scaffolds of MAO-B inhibitors and their clinical outcomes.

Scaffold	Mode of Action	Compound	Preclinical and Clinical Trial/s
**Synthetic** **propargyl phenyl analog of alkyl amine**	Irreversible selective MAOB inhibitor	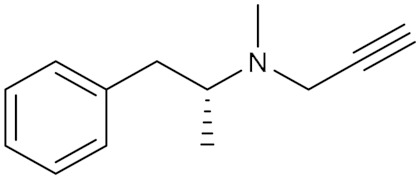 Selegiline (**1**) IC_50_ = 20 nM Ki = 0.5 nM	In vitro and in vivo studies [15] Phase II clinical trial ID: NCT04586543 [24]
		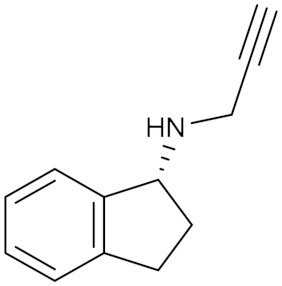 Rasagiline (**2**) IC_50_ = 4.4 nM Ki = 0.2 nM	In vitro and in vivo studies [15]
		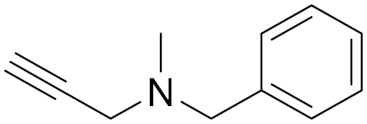 Pargyline (**3**) IC_50_ = 8.2 nM Ki = 5 nM	In vitro studies [13,68]
**Synthetic** **cyclopropylamine** **derivative**	Irreversible Non-selective MAO A/B inhibitor + LSD1 inhibitor	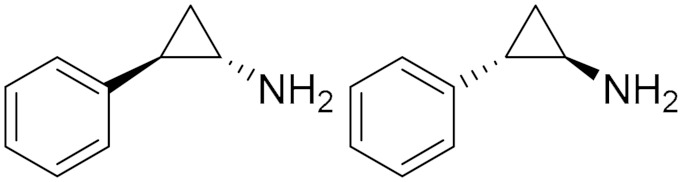 Tranylcypromine TCP (**4**) IC_50_ = 0.95 µM Ki = 10 nM	Phase I clinical trial ID: NCT02273102 [22] Phase I/II clinical trial ID: NCT02261779 [23]
**Synthetic** **hydrazine** **derivative**	Irreversible Non-selective MAO A/B Inhibitor	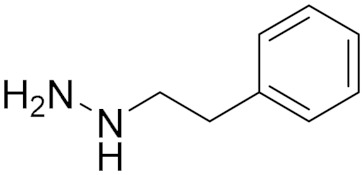 Phenelzine (**5**) IC_50_ = 0.9 μM Ki = 50 nM	In vivo studies [14] Phase Ib clinical trial ID: NCT03505528 [25] Phase II clinical trial, ID: NCT02217709 [17] Phase II clinical trial, ID: NCT01253642 [21]
**Natural** **phenylpropanoid derivative**	Nonselective MAO A/B inhibitor	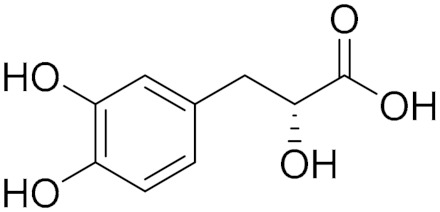 Danshensu (**6**) IC_50_ = 8.3 μM Ki = 34 μM	In vitro and in vivo studies [12]
**Prodrug** **Synthetic** **pyridine derivative linked to N-mustard**	Irreversible MAOB binding	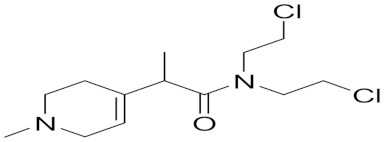 MP-MUS (**7**) IC50 = 80 μM	In vitro studies [69]
**Polyamine** **Derivatives**	Nonselective MAO (A/B) inhibitor	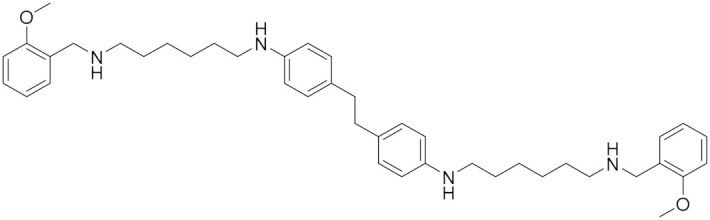 Compound (**14**) IC_50_ = 0.9 μM Ki = 0.3 μM. 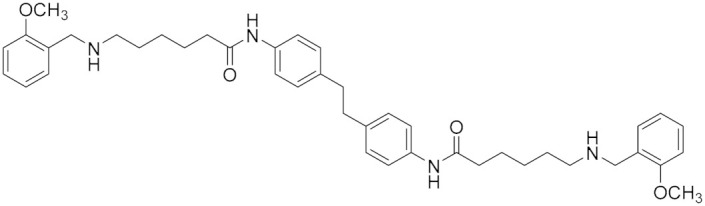 Compound (**15**) IC_50_ = 0.8 μM Ki = 0.2 μM	In vitro studies [5]
**Synthetic Hydrazothiazole Derivatives**	Irreversible selective MAOB inhibitor	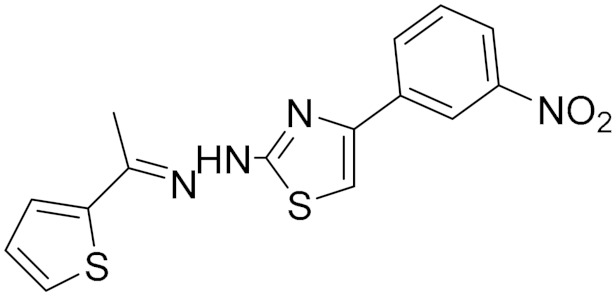 Compound (**16**) IC_50_ = 0.0068 μM Ki = 6.8 nM 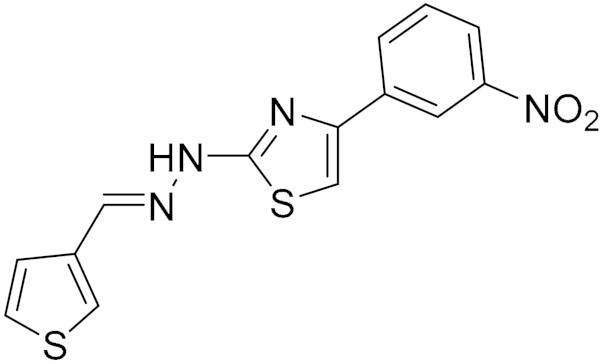 Compound (**17**) IC_50_ = 0.0025 μM Ki = 2.5 nM	In vitro studies [11]
**Synthetic Chalcone** **Derivatives**	Irreversible selective MAOB inhibitor	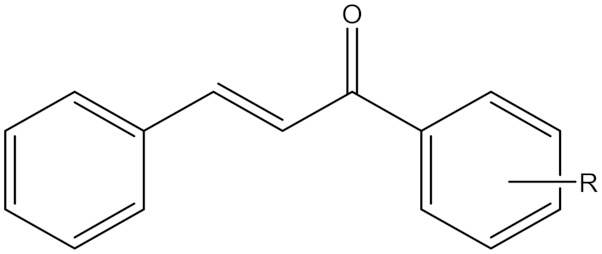 R = *m*-CF3, Compound (**19**) R = *p* -CF3, Compound (**20**) IC_50_(**19**) = 5 nM Ki (**19**) = 5.0 nM IC_50_(**20**) = 14.6 nM Ki (**20**) = 14.6 nM	In vitro studies reference [19]
**Synthetic Chromone** **Derivatives**	Irreversible selective MAOB inhibitor	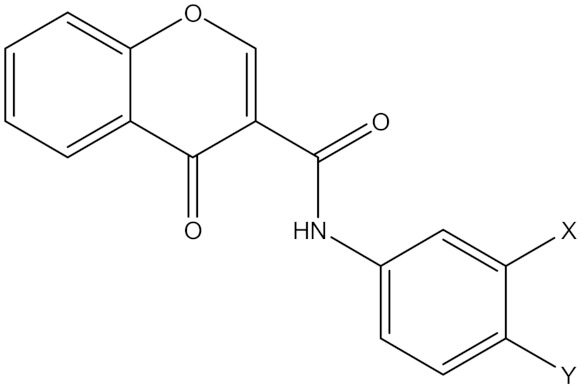 Compound (**21**), X = CH_3_, Y = CH_3_ Compound (**22**), X = CL, Y = H K_i_ (**21**) = 55.0 nM K_i_ (**22**) = 17 nM	In vitro studies [20]

Kormos et al. revealed that selegiline (**1**) and rasagiline (**2**) significantly reduced the viability of an androgen-independent human prostate cancer cell line PC3 and an androgen-dependent human prostate cancer cell line LNCaP in a concentration-dependent manner [15]. A follow-up in vivo experiment conducted for (**1**) and (**2**) on a human PC3 xenograft model using an NSG SCID mouse showed that both compounds induced a significant reduction in the rate of tumor growth and diminished the prostate volume over the treatment time [15].

A combination of selegiline (10 mg daily) with the standard regimen of chemotherapeutic agent carboplatin for the treatment of male drug with grade II–III prostate cancer for 150 days provides a significant improvement in the clinical parameter (urinary symptoms, prostate volume, and pain-free condition) compared to carboplatin alone [15]. In recent clinical studies, selegiline was added to the regimen of chemotherapeutic agents to evaluate its add-on efficacy and safety. An ongoing phase 2 clinical trial for assessing the efficacy and safety of using selegiline with docetaxel for the treatment of patients diagnosed with metastatic, castrate-resistant prostate adenocarcinoma has been conducted, and the result will be accessible by 2025 [24].

Pargyline (**3**) (N-methyl-N-2-propynylbenzene-methylamine), is a selective and irreversible MAO-B inhibitor that was discovered and evolved as an antihypertensive agent. Its impact is supposed to be facilitated by the suppression of norepinephrine deamination. It was brought to the market in 1963 but terminated later in 2007 because of its moderate to severe interaction with many other drugs and foods [67].

Pargyline (**3**) was found to decrease the growth of T47D human breast cancer cells in a dose-dependent manner; this impact was linked to an increase in the G1 phase and a decrease in the S phase. Moreover, it increases T47D apoptosis, leading to an increase in cleaved poly (ADP-ribose) polymerase (PARP), a class of proteins involved in genetic integrity, DNA repair, and programmed cell death, among other cell cycle processes [13].

In a subsequent study, Lee et al. studied the effect of pargyline (**3**) and tranylcypromine (**4**) on cell viability in the human prostate carcinoma (LNCaP-LN3) cell line. The multiplication of cells exposed to pargyline diminished significantly in time and pattern compared to the control and tranylcypromine-exposed cells. Pargyline significantly triggers cell cycle arrest in the G1 phase and increases the cell death rate by fostering apoptosis. The latter effect was related to the observed increment of the expression of the NOXA apoptosis-related gene and decreasing the expression of the anti-apoptotic protein BCL-2 [68].

### 5.2. Cyclopropylamine Derivatives

Tranylcypromine (**4**), also known as TCP and 2-PCPA, belongs to the class of irreversible, non-selective cyclopropylamine-based MAO inhibitors. In 1961, tranylcypromine was approved by the Food and Drug Administration (FDA) as an antidepressant drug that has been utilized for patients with major depressive disorder due to the ability to increase neurotransmitter levels in the brain by inhibiting the catabolism of serotonin and norepinephrine [11]. Following the repurposing strategy of antidepressant drugs in the treatment of cancer, tranylcypromine has progressed into clinical trials for cancer therapy [70,71]. Research has shown that tranylcypromine has antitumor activity as it functions as a dual inhibitor of both MAOs and LSD1 [72]. The IC_50_ of tranylcypromine is 2.3 µM, 0.95 µM, and 20.7 µM against MAO-A, MAO-B, and LSD1, respectively [73]. Moreover, further medicinal chemistry efforts with tranylcypromine have led to the discovery of several tranylcypromine-related molecules for their use as potential antitumor agents with inhibitory activity in vitro against both MAOs and LSD-1 [2].

Tranylcypromine showed efficacy against glioblastoma and squamous cell carcinoma of the head and neck [2]. In 2023, Chen et al. studied the effect of using a combination of tranylcypromine and ML385 (an NRF2 inhibitor) into Nrf2 knockout cells, which resulted in a significant reduction in tumor proliferation, although neither Tranylcypromine nor Nrf2 knockout alone had a significant effect on cancer cell growth [72].

Two clinical trials were conducted to assess the efficacy and safety of using a combination of TCP with the retinoid all-trans-retinoic acid (ATRA) for the treatment of patients with acute promyelocytic leukemia (non-APL) subtype of acute myeloid leukemia (AML) that is resistant to ATRA therapy, unlike the other APL. It was found that TCP could re-sensitize non-APL AML cells to ATRA with reasonable clinical activity and safety [22,23]. Later investigations by Tayari et al. demonstrated that TCP boosts the expression of ATRA-responsive genes and so restores its antileukemic activity. In addition, the combination therapy could revitalize *Ragene* expression that controls the differentiation of immature white blood cells, which was quiescent in non-APL AML ATRA-resistant patients [74].

### 5.3. Hydrazine Derivatives

Phenelzine (**5**) (also known as Nadril) belongs to the class of non-selective and irreversible hydrazine derivative MAO inhibitors. To date, it is indicated for the treatment of non-endogenous, neurotic, or atypical depression in patients who do not respond to other drugs [75]. Additionally, compound (**5**) has been reported in clinical studies to be effective in the treatment of anxiety disorders such as panic disorder and social anxiety disorder [75].

Moreover, phenelzine has shown promising activity in different clinical trials for treating prostate and metastatic breast cancer. As monotherapy, it revealed effectiveness in patients with biochemical recurrent castrate-sensitive prostate cancer [17]. Also, phenelzine sulfate acts synergistically with docetaxel in prostate cancer patients who have evidence of progression on standard docetaxel therapy. This combination was well tolerated without significant toxicity [21]. Phenelzine was proposed for treating bone metastases in prostate cancer in combination with 4-aminoquinoline and tyrosine kinase inhibitors [14].

Phenelzine showed another proposed anticancer activity by inhibiting lysine-specific demethylase-1 (LSD-1). A study conducted by Prasanna et al. showed that phenelzine and nab-paclitaxel showed evidence of antitumor activity in patients with metastatic breast cancer. In vivo inhibition of LSD-1 reactivated the tumor suppressor gene that the cancer scells rely on for growth and survival and impeded the generation of cancer stem cells with metastatic capabilities [76].

### 5.4. Natural Phenylpropanoid Derivative

Danshensu (DSS) (**6**), (3-(3,4-dihydroxyphenyl)-2-hydroxy-propanoic acid) is a hydrophilic active component of danshen; the dry root and rhizome of the herbaceous plant called *Salvia miltiorrhiza* Bunge. Danshensu was first known for its ability to inhibit platelet aggregation and for its antioxidant properties [77]. NSCLC, which represents 85% of lung cancers, is a challenging cancer type with a high rate of mortality due to late-stage diagnosis and rapid emergence of resistance to chemotherapy and radiation therapy. Using traditional oriental medicine (TOM) as adjuvant therapy in NSCLC patients has been reported to improve the efficacy and sensitivity of chemotherapy and radiation therapy in treating this type of cancer [18].

Depending on a target-based approach for several enzymes (especially MAO-B due to its overexpression in cancerous lung tissue), Son et al. screened many TOM-derived compounds as candidate potential radio-sensitizing agents for NSCLC. Danshensu showed a significant affinity for MAO-B and inhibited its enzyme activity by 50% (IC_50_ = 8.3 μM) via direct binding. MAO-B activity could relieve radio resistance by inactivating NF-κB, which is overexpressed in many cancer types that show resistance to chemotherapy and radiotherapy [12].

The NF-κB-specific luciferase reporter assay indicated that the IR-induced transcriptional activation of NF-κB was significantly reduced by treatment of human lung adenocarcinoma cell line (A549) and NCI-H1299 cells (NSCLC cell lines) with 2 Gy of IR plus danshensu (50 μM), selegiline (2 μM), or MAO-B siRNA. As compared with untreated NSCLC cells, danshensu-treated cells were more sensitive to IR-mediated cytoplasmic histone-associated DNA fragmentation, a measure of apoptotic cell death. On the other hand, morphological modifications showed that IR-induced Epithelial-Mesenchymal Transition (EMT) was inhibited by danshensu and selegiline, as well as cell mobility assessed by cell migration and wound healing assays [77].

In vivo experiments on nude mice xenograft model affected with tumors formed by A549 or NCI-H1299 cells showed that the tumor volumes of mice treated with IR and danshensu were significantly reduced by 30.3% (for A549 cells) or by 27.9% (for NCI-H1299 cells) at 30 days (about 4 and a half weeks) as compared with mice treated with IR alone [12].

### 5.5. Synthetic Pyridine Derivative Linked to Nitrogen Mustard

Glioma is the prevailing type of brain cancer in humans. It is a poorly prognostic tumor that is resistant to many existing therapies. Histological studies demonstrate a significant and selective increment in MAO-B activity in human gliomas when compared with other types of brain tumors or normal brain tissue [11]. MAO-B is primarily located on the inner side of the mitochondrial outer membrane of the glioma cells [71].

Sharpe et al. utilized this distinct feature in designing the MP-MUS prodrug (**7**) that uses MAO-B as a catalyst for bioconversion into an active mitochondrial targeting chemotherapeutic agent [69].

**Figure 4:**
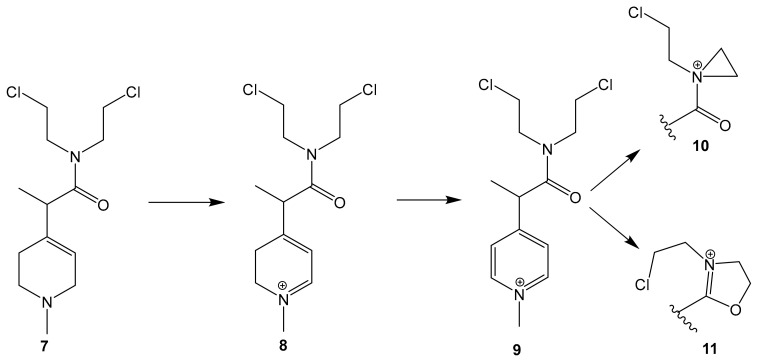
MP-MUS MAO-B dependent bioactivation pathway.

MP-MUS (**7**) is a chimeric MPTP (1-methyl-4-phenyl-1,2,3,6-tetrahydropyridine) Nitrogen Mustard drug. It is oxidized selectively by MAO-B, forming the intermediate MP-MUS+ (**8**) that is oxidized to the active constituent, P+-MUS (**9**). The formed lipophilic cation P+-MUS will stack in the mitochondrial matrix of cancerous glial cells, driven by its high membrane potential [78]. Nitrogen mustards alkylate mitochondrial DNA and ribosomal RNA via the formation of a highly reactive three-membered aziridinium ring (**10**) and the dihydrooxazolium cationic ring (**11**) that formed in the presence of an amide group in MP-MUS/P+ (Figure 4). Mitochondrial DNA alkylation leads to mitochondrial malfunction and cell death [79].

MP-MUS demonstrated significant cell toxicity on primary glioma cells as indicated by the XXT assay, alongside the significant decrease in mitochondrial membrane potential and a notable increase in LDH and ROS levels. The LD_50_ was 77 μM after 24 h of exposure. MP-MUS treatment was also successful in lowering the glioma burden in primary human glioma xenografts in a Nu/Nu nude mouse model. A single treatment of 8 mg/kg MP-MUS was able to shrink tumor volume by 50% after 24 h of exposure [69].

Co-administration of MP-MUS with the MAO-B-specific inhibitor selegiline resulted in the attenuation of MP-MUS mitochondrial toxicity; this observation confirmed the MAO-B-dependent bioactivation pathway [80].

### 5.6. Polyamine Derivatives

**Figure 5:**
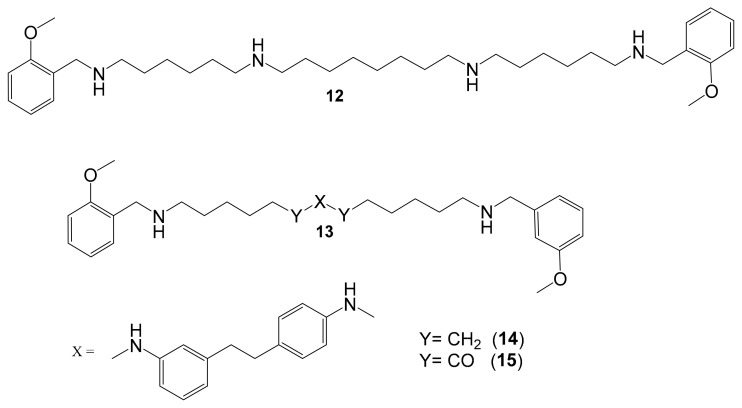
Polyamine analogs. Compounds **14** and **15** have remarkable anti-proliferative activity in LN-229 (GI_50_ < 1 µM) while compound **12** has GI_50_ > 20 µM. Compound **13** is structurally related to **12**.

The polyamine skeleton represents a universal template as the insertion of appropriate moieties on amine groups and the type of linker between them can modulate selectivity and affinity toward a given receptor or enzyme [81]. Some polyamine-based analogs can inhibit amine oxidases, such as human MAOs [82]. Emanuela et al. (2012) have demonstrated a decrease in the flexibility of the inner polymethylene chain of methoctramine (**12**) and its polyamine-based analogs (**13**) such as the di-piperidine analog, resulting in an active and reversible MAO B inhibitor [82]. In 2023, Nordio et al. reported several polyamine analogs (**13**) that are structurally related to compound (**12**) and are characterized by constrained linkers between the inner amine functions of the polyamine backbone (Figure 5) [5]. Considering the overexpression of MAOs in glioblastoma and the effects of MAOIs in glioma progression, the role of these analogs on the MAO inhibitory activity and the anti-proliferative activity were investigated. These analogs are distinguished by constrained linkers between the inner amine functions of the polyamine backbone. It was verified that a decrease in the flexibility of the inner polymethylene chain of methoctramine (compound **12**) is favorable to MAO B inhibitory activity. Two of these analogs (**14** and **15**) were found to join these activities, exhibiting remarkable anti-proliferative activity in LN-229, a human glioblastoma cell line (GI_50_ < 1 µM) with almost by more than two orders of magnitude with respect to compound **12**, noting that compound **12** was used as a reference having GI_50_ > 20 µM [5].

### 5.7. Synthetic Hydrazothiazole Derivatives

**Figure 6:**
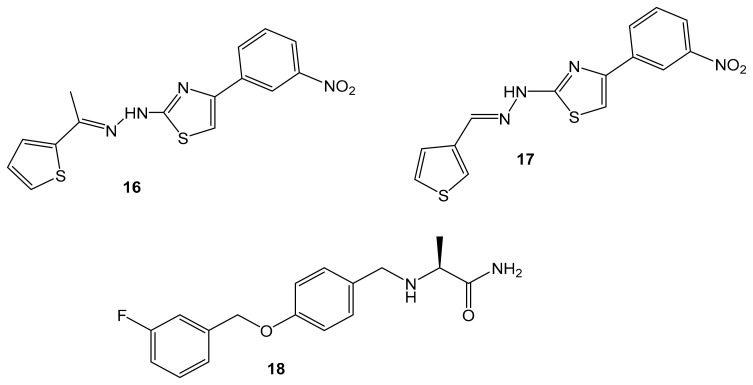
Structures for compounds (**16**–**18**).

In 2019, Marconi and colleagues evaluated the effectiveness of two derivatives of the hydrazothiazole scaffold synthesized by Secci and colleagues in the treatment of glioma [11,83]. These derivatives, compound (**16**) and compound (**17**) (Figure 6) have potent and selective hMAO-B inhibition in the low nanomolar range. Compound (**16)** had an IC_50_ value of 0.0068 μM, while compound (**17**) had an IC_50_ value of 0.0025 μM, and they both were shown to act as reversible and competitive inhibitors [11]. This was compared with two approved selective MAO-B inhibitors, selegiline (**1**) and safinamide (**18**). Safinamide acts through a dual mechanism; it combines dopaminergic effects as it is highly selective and reversible MAO-B inhibitor action [84] along with non-dopaminergic properties by blocking voltage-dependent sodium channels and modulating calcium channels [85]. However, safinamide was completely ineffective on human cancer cells [19].

Marconi and colleagues conducted a study to investigate the biological response of rat C6 glioma cell line and CTX TNA2 astrocytes after treatment with the two novel MAO-B inhibitors (**16**) and (**17**) by evaluating cell proliferation, apoptosis occurrence, inflammatory events, and cell migration [11]. These two novel MAO-B inhibitors have been shown to inhibit glioma proliferation by arresting the cell cycle and significantly increasing oxidative stress conditions while reducing the invasiveness of malignant cells by slowing their migration. These results support the critical role played by MAO-B in mediating oncogenesis in glioma. Thus, targeting the MAO-B protein could be a novel approach to achieving improved therapeutic efficacy for glioblastoma [11].

### 5.8. Synthetic Chalcone Derivatives

Chalcones are open-chain flavonoids containing benzylideneacetophenone scaffolds where two aromatic nuclei (A and B ring) are joined through an α, β-unsaturated carbonyl linker [19,86]. This unique chemical structure empowers chalcones with the capability to hit different targets (enzymes and receptors) and exert a variety of biological activities. The chalcone structure motif is therefore widely used in medicinal chemistry and drug discovery to obtain derivatives with anticancer, antioxidant, antiviral, and anti-inflammatory activities and for the treatment of neurodegenerative disorders [19].

The majority of chalcones exhibit selective, reversible, and potent MAO-B inhibition compared to MAO-A. Such compounds have shown MAO-B inhibitory activity in the nanomolar range and also good selectivity, making them potential anticancer agents [86]. Unsubstituted chalcone scaffold induced a significant antiproliferative effect, with GI_50_ values ranging from 2.8 to 16.7 μM [19]. Additionally, various group substitutions determine the MAO inhibitory activities of natural and synthetic chalcones [19,86].

A study done by Iacovino et al. revealed that different chalcone series result in dissimilar antiproliferative activity. In this study, an in vitro assay was performed on three tumor cell lines, i.e., A2780 (ovarian carcinoma), HT-29 (colorectal adenocarcinoma), and MSTO-211H (biphasic mesothelioma). It revealed that GI _50_ values of compound (**19**) were 10, 12.7, and 4.5 μM towards A2780, HT-29, and MSTO-211H cell lines, respectively. On the other hand, the GI _50_ values of compound (**20**) obtained were 14, 12.2, and 7 μM on all the previously considered cell lines. It is worth mentioning that inhibition constant values for compounds (**19**) and (**20**) on MAO B are 5 nM and 14.6 nM. Meanwhile, the selectivity index (Ki(MAO-A)/Ki(MAO-B)) is 920:1 for compound (**19**) and 630:1 for compound (**20**) [19].

### 5.9. Synthetic Chromone Derivatives

A study reported by Reis et al. highlighted the biological evaluation of chromones (compounds **21** and **22**) on ROS production in HEK 293 cells transfected with human MAO B and stimulated with the substrate tyramine [20]. The specific involvement of MAOB in ROS generation was supported by the complete prevention of DFCDA production by the MAO-B inhibitor L-deprenyl. Interestingly, an observed in the cellular context with a 1000-fold stronger reduction of ROS levels in treated HEK-293 cells for both compounds (**21**) and (**22**) compared to the well-known covalent MAO-B inhibitor L-deprenyl (*p* < 0.01). These findings make chromone a valuable scaffold for the development of novel potent and reversible inhibitors of human MAO-B [20]. Ki values of compounds (**21**) and (**22**) are 55 and 17 nM, respectively [20].

## 6. Conclusions

In the search for new anticancer medicines, targeting MAO-B with small-molecule inhibitors has shown great promise. According to experimental research, a favorable environment for the initial growth and spread of tumors may be generated by MAO-B’s ability to produce ROS through its oxidative deamination activity. Numerous cancer types, including colorectal, glioblastoma, breast, lung, pancreatic, and liver cancers, have overexpressed MAO-B, which reinforces the suggested connection. Several MAO-B inhibitor scaffolds have been identified as potential anticancer agents that, in the preclinical stage, clearly affect cellular proliferation and apoptosis. MAO-B inhibitors that have successfully reached phase I and phase II clinical trials proved capable of improving the results of cancer treatment. In this review, we demonstrated how MAO-B metabolic function is related to the onset and spread of cancer, and we present the MAO-B inhibitors under investigation that have shown anticancer activity in preclinical and clinical settings. In addition, we provided systems biology evidence that MAO-B is involved in key glycolysis pathways that may contribute to ROS-induced tumorigenesis. Moreover, the structural analysis of MAO-B ligand interactions revealed a relatively hydrophobic pocket with some key polar residues such as Tyr398 and Tyr435 that play crucial roles in substrate oxidation.

## Data Availability

Not applicable.

## Supplementary Materials

The following supporting information can be downloaded at: https://www.mdpi.com/article/10.3390/molecules30010126/s1. Table S1. Enrichment results for the direct interactions network of MAO-B. Table S2. Enrichment results for the expanded interactions network of MAO-B.

## Abbreviations

**Order Regarding** **Appearance in Text**:	**Abbreviation**	
1	MAO-B	Monoamine oxidase B
2	ROS	Reactive oxygen species
3	MAO	Monoamine oxidase
4	FAD	Flavin adenine
5	MAO-A	Monoamine oxidase A
6	CNS	Central nervous system
7	ALDH	Aldehyde dehydrogenase
8	H_2_O_2_	Hydrogen peroxide
9	NH_3_	Ammonia
10	FADH_2_	Dihydro-flavin adenine dinucleotide
11	R-CH_2_-CH_3_	Amine neurotransmitter
12	O_2_	Oxygen
13	BC	Bladder cancer
14	WHO	World Health Organization
15	HIF-1α factor	Hypoxia-inducible factor 1
16	VEGF	Vascular endothelial growth factor
17	NF-kB	Nuclear factor kappa B subunit 1
18	CRC	Colorectal cancer
19	BGA	Brain-gut axis
20	ENS	Enteric nervous system
21	LX-2	Hepatic stellate cells
22	GGOH	Geranylgeraniol
23	GGA	Geranylgeranoic acid
24	siRNAs	Small interfering RNA
25	NSCLC	Non-small cell lung cancer
26	IR	Ionizing radiation
27	RCC	Renal cell carcinoma
28	ccRCC	Clear cell renal cell carcinoma
29	NN	Nearest neighbor
30	GLUT1	Glucose transporter protein type 1
31	HK2	Hexokinase 2
32	SOD	Superoxide dismutase
33	PARD	Poly (ADP-ribose) polymerase
34	LSD-1	Lysine specific demethylase-1
35	TCP	Tranylcypromine
36	ATRA	Retinoid all-Trans-Retinoic Acid
37	AML	Acute myeloid leukemia
38	DSS	Danshensu
39	TOM	Traditional Oriental Medicine
40	PARP	Poly ADP-ribose polymerase
41	FDA	Food and Drug Administration
42	MP-MUS	1-Methyl-4-Phenyl-1,2,3,6-tetrahydropyridine-nitrogen Mustard
46	A2780	Ovarian carcinoma
47	HT-29	Colorectal adenocarcinoma
48	MSTO-211H	Biphasic mesothelioma
